# *Post hoc* Correction of Chromatic Aberrations in Large-Scale Volumetric Images in Confocal Microscopy

**DOI:** 10.3389/fnana.2021.760063

**Published:** 2021-12-10

**Authors:** Marcus N. Leiwe, Satoshi Fujimoto, Takeshi Imai

**Affiliations:** Department of Developmental Neurophysiology, Graduate School of Medical Sciences, Kyushu University, Fukuoka, Japan

**Keywords:** chromatic aberration, tissue clearing, volumetric imaging, multicolor imaging, confocal microscopy, connectomics

## Abstract

Over the last decade, tissue-clearing techniques have expanded the scale of volumetric fluorescence imaging of the brain, allowing for the comprehensive analysis of neuronal circuits at a millimeter scale. Multicolor imaging is particularly powerful for circuit tracing with fluorescence microscopy. However, multicolor imaging of large samples often suffers from chromatic aberration, where different excitation wavelengths of light have different focal points. In this study, we evaluated chromatic aberrations for representative objective lenses and a clearing agent with confocal microscopy and found that axial aberration is particularly problematic. Moreover, the axial chromatic aberrations were often depth-dependent. Therefore, we developed a program that is able to align depths for different fluorescence channels based on reference samples with fluorescent beads or data from guide stars within biological samples. We showed that this correction program can successfully correct chromatic aberrations found in confocal images of multicolor-labeled brain tissues. Our simple *post hoc* correction strategy is useful to obtain large-scale multicolor images of cleared tissues with minimal chromatic aberrations.

## Introduction

**FIGURE 1:**
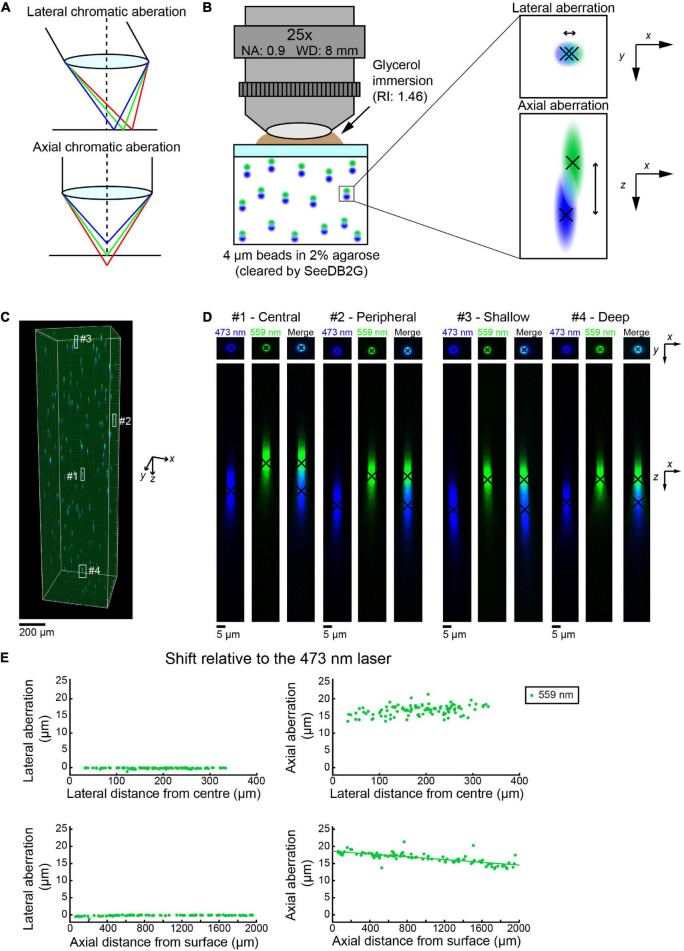
Measuring chromatic aberrations with fluorescence beads in SeeDB2G (Olympus 25×). **(A)** Lateral and axial chromatic aberrations can occur in confocal microscopy. **(B)** Measurement of chromatic aberrations with a 25× custom-made objective lens (Olympus). Fluorescent beads (4 μm) were embedded in agarose gel and then cleared with SeeDB2G (RI, 1.46). The agarose sample was mounted with a 0.17 mm thick coverslip. Glycerol (RI, 1.46) was used for immersion. **(C)** A volumetric confocal image of the fluorescent beads in the gel (volume rendering). **(D)** High-magnification images of representative beads [*x–y* and *x–z* maximum intensity projections (MIPs)]. Centroid positions for each channel are shown by black crosses. **(E)** Lateral and axial chromatic aberrations at 559 nm were determined as shifts relative to the 473 nm laser. Positive axial aberration values indicate that the bead is higher than it appears in the 473 nm image. Axial aberrations were more pronounced. Moreover, the axial aberrations were depth-dependent and could be fit with a regression line (green line).

Combined with genetically encoded fluorescent markers, fluorescence imaging is a powerful strategy for mesoscopic mapping of neuronal circuits. During the past decade, various types of tissue clearing agents compatible with fluorescent proteins have been developed (Richardson and Lichtman, 2015). This expanded the imaging scale to a millimeter scale. A major advantage of fluorescence imaging is multicolor imaging. By using different combinations of excitation and emission ranges, we can easily obtain images from different fluorophores separately and then merge them to make composite images. For example, in one application, a combination of only three types of fluorescent proteins can produce various color combinations in neurons, and that can be used to trace numerous neurons within a densely labeled neuronal circuit (Lichtman et al., 2008). However, multicolor imaging of large volume faces technical challenges. One of the most notable challenges is chromatic aberration. Chromatic aberration occurs as different wavelengths of light refract at different angles depending on the substance it is passing through. This can produce two different types of chromatic aberrations, namely, lateral (displacement along the *x-* and *y*-axis) and axial (displacement along the *z*-axis, Figure 1A). When tracing fine structures such as neurites and synapses with multiple fluorophores, this distortion can lead to a difference in the position.

This problem has been well documented and analyzed for the past 300 years of microscopy, and there are many lenses that “fix” chromatic aberration issues by using a combination of crown and flint glasses. For example, the PLANAPO by Olympus and the Apochromats from Leica produce reduced chromatic aberrations.

However, these corrections are still imperfect when imaging cleared tissues. Currently, there are various types of clearing agents, and the refractive index of the clearing agent is not necessarily the same as the immersion media. A refractive index mismatch can easily produce chromatic aberrations. Indeed, matching the refractive index of the immersion and clearing agents is essential to minimize spherical aberration, as has been fully discussed by Ke et al. (2016) and Ke and Imai (2018). Also, the correction collar should be used to minimize the spherical aberrations.

Even when the refractive index of the clearing agent is exactly the same as the immersion medium at a defined wavelength, the Abbe numbers may be different between clearing agent and immersion medium. Therefore, chromatic aberrations are almost inevitable when imaging cleared tissues with different wavelengths of light. This means that *post hoc* processing is needed to realign images produced from different wavelengths of light. The best way to record these differences is through the use of fluorescent beads that are excited by and emit different wavelengths of light. By isolating a single bead, the distortions between different channels can be recorded and then applied to any images acquired under the same settings.

There are many freeware applications, such as MetroloJ (Mathhews and Cordeliers, 2010), that can measure these changes and therefore allow the experimenter to correct these distortions at a defined position. Yet, these applications are only able to measure one bead at a time and do not take into account the location of the beads within the field and depth of view. Previously, the *post hoc* correction of chromatic aberrations (i.e., pixel shifts) has been performed only uniformly to the entire volume (Bogovic et al., 2020; Reitz et al., 2021). To fix the distortions within the entire image volume accurately, we need to know the distribution of the distortion at different locations within the volume. This will be particularly important for large volumetric samples.

In this study, we evaluated chromatic aberration for different objective lenses and confocal microscopes using fluorescent beads embedded in cleared agarose gels. Then, we performed linear regressions to calculate what transformations are needed to align fluorescence images taken with different excitation lasers to each other. Finally, we picked one excitation laser as a reference and applied the relevant transformations to obtain aberration-corrected volumetric images of multicolor-labeled neuronal circuits.

## Methods

All experiments were conducted in compliance with the biosafety and institutional safety guidelines of Kyushu University. Animal experiments were approved by the Institutional Animal Care and Use Committee (IACUC) of Kyushu University.

### Bead Preparation

A total of 10 μl of Tetraspeck beads (200 nm, 500 nm, or 4 μm, ThermoFisher Cat#T14792) were diluted into 1 ml of 2% UltraPure low-melting-point agarose (ThermoFisher, Cat#16520-100). The solution was then vortexed and poured into a mold and left at 4°C to solidify into a block. This block was then sectioned using a vibratome (Linear Slicer Pro7, DSK, Japan, Cat#PRO7) into 300 μm or 2 mm section and prepared for clearing or mounting as described in Ke and Imai (2018). One block was prepared for each imaging condition.

### *In utero* Electroporation

*In utero* electroporation was performed as described previously (Sakaguchi et al., 2018). To label cortical layer 2/3 neurons with multiple colors, ∼2 μl of Tetbow plasmids (0.1 μg/μl of pCAG-tTA RRID:Addgene_104102, 0.25 μg/μl of pBS-TRE-mTurquoise2-WPRE RRID:Addgene_104103, 0.25 μg/μl of pBS-TRE-EYFP-WPRE, RRID:Addgene_104104, and 0.25 μg/μl of pBS-TRE-tdTomato-WPRE RRID:Addgene_104105) (Sakaguchi et al., 2018) were injected into the lateral ventricle at E15 and electric pulses (a single 10 ms poration pulse at 72 V, followed by five 50 ms driving pulses at 42 V with 950 ms intervals) were delivered along the medio-lateral axis of the brain with forceps-type electrodes (5 mm diameter, BEX, Cat# LF650P5) and a CUY21EX electroporator (BEX). Mice were analyzed at P25 or P101. All animal experiments were approved by the Institutional Animal Care and Use Committee of Kyushu University (#A21-117-1) and were carried out in accordance with the regulation and guidelines for the care and use of experimental animals at the Kyushu University.

### Clearing

Brain or bead samples were cut at 2 mm (for Olympus 25×) or 300 μm (for Leica 63× and 20×) thickness. Bead samples were then optically cleared using the SeeDB2G method as described previously (Ke and Imai, 2018). When correcting chromatic aberration using the regressions from beads, samples were cleared using the SeeDB2G protocol. However, for the guide star evaluation, clearing was performed with the SeeDB2S protocol in order to improve the resolution. Omnipaque 350 (Daiichi-Sankyo) or Histodenz (Sigma-Aldrich) was used to prepare SeeDB2G/S solutions.

### Confocal Imaging

Confocal images were obtained using either a confocal/two-photon microscope (Olympus FV1000MPE with Fluoview FV10-ASW, RRID:SCR_014215) or an inverted confocal microscope (TCS SP8 with HyDs, Leica Microsystems, RRID:SCR_002140). In the FV100MPE, EYFP and tdTomato were excited with the 473 and 559 nm lasers, respectively, and were separated by a GFP/RFP filter set. In TCS SP8, mTurquoise2, EYFP, and tdTomato were excited with 405, 488, and 552 nm, respectively. Emission signals were dispersed by a diffraction gating and detected by HyD detectors.

For deep imaging with the Olympus confocal microscope, a custom-made 25× objective lens optimized for high-index samples was used (Olympus, NA 0.9, WD 8 mm, RI 1.41–1.51). A similar commercially available lens is available from Olympus as XLSLPLN25XGMP. Confocal images were acquired at 1 A.U.

For imaging depths down to 300 μm, the inverted confocal microscope set up was used with one of the two objectives, i.e., a 20× multi-immersion objective lens (Leica Microsystems, HC PL APO 20x/0.75 IMM CORR CS2, NA 0.75, WD 0.66 mm) or a 63× glycerol-immersion lens (Leica Microsystems, HC PL APO 63x GLYC CORR CS2, NA 1.3, WD 0.28 mm). Both the 20× and 63× correction collars were set to match the glycerol immersion medium. Confocal images were acquired at 1 A.U. for bead images. Only for Figures 5D–F with the 63× objective lens, confocal images were acquired at 0.6 A.U., and images were further processed by LIGHTNING (Leica Microsystems, RRID:SCR_018169) to obtain deconvoluted superresolution images.

### Image Analysis

All MATLAB codes written for this project are available at the following link, along with a Readme instruction^1^. Raw images and corrected images from additional samples are available^2^. For the Olympus ×25, one image was provided from one animal, for the 20× and 63× Leica objectives, four images were evaluated from four mice.

#### Bead Detection

Beads were selected by manually tracing rectangular regions of interest (ROIs) from a maximal intensity projection along the *z* depth in Fiji [NB: Care was taken to make sure that each ROI contained only a single bead (Schindelin et al., 2012), RRID:SCR_002285]^3^. Alternatively, the beads were detected using the spot detector available in ICY (Olivo-Marin, 2002) (RRID:SCR_010587).

Within the codes provided, there is also the possibility of auto-detecting the beads using a threshold-based metric.

#### Chromatic Aberration Measurement

Voxels from the ROI for each bead were used. To calculate the position of the bead in each channel, the center of mass was calculated. According to the following formula,


$$CenterofMass\left[x,y,z\right]=\left[\sum_{i=1}^{nVx}\frac{I_{Vx_{i}}*Vx_{x}}{\sum I_{Vx}},\sum_{i=1}^{nVx}\frac{I_{Vx_{i}}*Vx_{y}}{\sum I_{Vx}},\sum_{i=1}^{nVx}\frac{I_{Vx_{i}}*Vx_{z}}{\sum I_{Vx}}\right]$$

where *I* is the intensity of the voxel *Vx* with its three coordinate locations, namely, *Vx*_*x*_, *Vx*_*y*_, and *Vx*_*z*_, and *nVx* is the total number of voxels.

To consider only genuine label, voxels with an intensity greater than the mean ± 10 standard deviations were considered, and shot noise was removed by median filtering along the *z*-axis by three voxels.

The distances between each center of mass were then calculated along each dimension.

#### Guide Star Measurements

Using FIJI, the raw 3D volume was loaded, and ROIs were selected where a neurite labeled with all fluorescent channels was present. Care was taken to make sure that for each guide star ROI, only a single triple-labeled neurite was included, i.e., no other neurites are present in the ROI. In addition, we made sure that for the *x–y* region selected, we included the full *z* extent of the neurite (otherwise, the center of mass calculations would be distorted). Then, each ROI was saved separately and then loaded into MATLAB for further analysis. In MATLAB, for each ROI, the center of mass was calculated, and the axial aberration was considered to be the distance in *z* between the center of mass for each channel.

Enough samples should be assured to produce an accurate regression for all combinations. The *post hoc* power analysis was performed in G*Power 3 (Faul et al., 2007, 2009); we derived Cohen’s *f*^2^ from the *r*^2^ values. If the power was over 0.7, we considered the regression to be sufficiently accurate. If not, then more guide stars were recorded until there was sufficient power.

#### 3D Visualization

For the qualitative assessment of 3D imaging volumes, orthogonal views were produced using FIJI. While for 3D visualization, the image data were downscaled by 50% and converted to 8 bit RGB images. These were then visualized using the Imaris 8.2 software (RRID:SCR_007370).

## Results

### Chromatic Aberrations in Volumetric Confocal Images of Cleared Samples

First, we evaluated chromatic aberrations in volumetric images of cleared samples. We embedded fluorescent beads (Tetraspeck beads, diameter: 4 μm) in 2% agarose gel and cleared the gel with a clearing agent, SeeDB2G (refractive index: 1.46) (Ke et al., 2016). Tetraspeck beads are excitable by four different wavelengths of light (i.e., 405, 473/488, 552/559, and 638 nm) in a confocal microscope, which enables us to measure the chromatic aberrations that occur when excited by different wavelengths (Figure 1B).

We used a custom-made 25× objective lens designed for cleared samples (Olympus, numerical aperture (NA) 0.9, working distance (WD) 8 mm, and the correction collar covers refractive index 1.41–1.51) (Ke et al., 2013) with an Olympus confocal microscope, FV1000 (Figure 1C). Initially, we chose to evaluate the differences between the two excitation lasers 473 and 559 nm by measuring the center of mass for multiple beads in all parts of a volume of 500 × 500 × 2,000 μm (black crosses, Figure 1D). Representative images show that the lateral chromatic aberration is minimal and almost unaffected by the *x–y* location of the beads in the image volume (center and peripheral beads, top panels, Figure 1D). The axial aberration was more pronounced in all the beads visualized and seemed to range from 15 to 20 μm along the *z* (depth)-axis (bottom panels, Figure 1E).

We measured the lateral and axial aberrations for 102 beads. The location of the bead was determined from the center of mass of the image from the 473 nm laser. The lateral and axial aberrations were measured relative to the image with the 473 nm laser. The lateral aberration is relatively small, i.e., <1 μm, across all distances from the center of the image plane (top left panel, Figure 1E). The lateral aberration was also unaffected by the depth of the bead (bottom left panel, Figure 1E). This indicates that we do not need to correct lateral aberrations in this imaging condition. In contrast, the axial aberration was consistently more pronounced. The image with the 559 nm laser was consistently shallower than the image produced by the 473 nm laser. Moreover, the axial aberration became smaller as the depth of the bead increases. A linear regression equation has been easily fitted to the plot (bottom right panel, Figure 1E). Therefore, using the equation, we should be able to retroactively transform the image with the 559 nm laser to fit the 473 nm image and produce an aberration-corrected image.

### Each Objective Lens Has a Unique Pattern of Chromatic Aberrations

The patterns of chromatic aberration can be different depending on the type of objective lens, laser line, and clearing agent. We, therefore, assessed two additional objective lenses, namely, a glycerin-immersion 63× (Leica, Microsystems, HC PL APO 63×/1.3 Gly CORR CS2, NA 1.3) and a multi-immersion 20× (Leica, HC PL APO 20×/0.75 IMM CORR CS2, NA 0.75). Here, we imaged Tetraspeck beads with four excitation lasers (i.e., 405, 488, 552, and 638 nm) using a Leica confocal microscope, TCS SP8.

**FIGURE 2:**
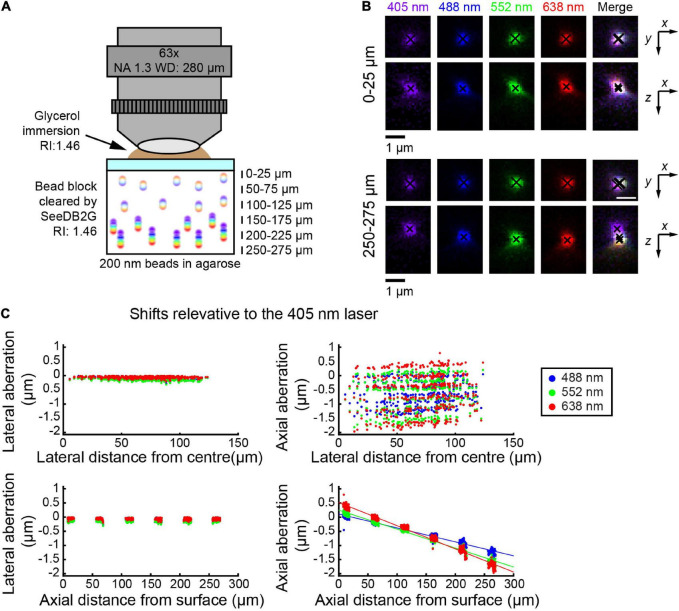
Measuring chromatic aberrations with a high-numerical aperture (NA) objective lens (Leica 63×). **(A)** Fluorescent beads (200 nm) were embedded in agarose gel and then cleared with SeeDB2G (RI, 1.46). The agarose sample was mounted with a 0.17 mm thick coverslip. Glycerol (RI, 1.46) was used for immersion. **(B)** High-magnification images of fluorescence beads at representative depths (MIPs). Centroid positions are indicated by black crosses. **(C)** Lateral and axial chromatic aberrations at 488, 552, and 638 nm were determined as shifts relative to the 405 nm laser. Positive axial aberration values indicate that the bead is higher than it appears in the 473 nm image. Depth-dependent axial aberrations were observed.

Using the glycerin-immersion 63× objective lens, we used smaller beads (i.e., 200 nm) and SeeDB2G (Figure 2A). By then, bead images were acquired at representative depths, till the upper limit of the WD. Again, the lateral aberrations were minimal, and more pronounced aberrations were found along *z*-axis (Figure 2B). At the depth of ∼50 μm, it appeared as if the chromatic aberrations are fully corrected both laterally and axially. When the shifts were calculated relative to the image with the 405 nm laser, axial aberrations were more prominent at deeper areas (bottom panels, Figure 2C). The pattern of chromatic aberrations was different from the 25× objective; however, linear regression lines were well fitted to the plots.

**FIGURE 3:**
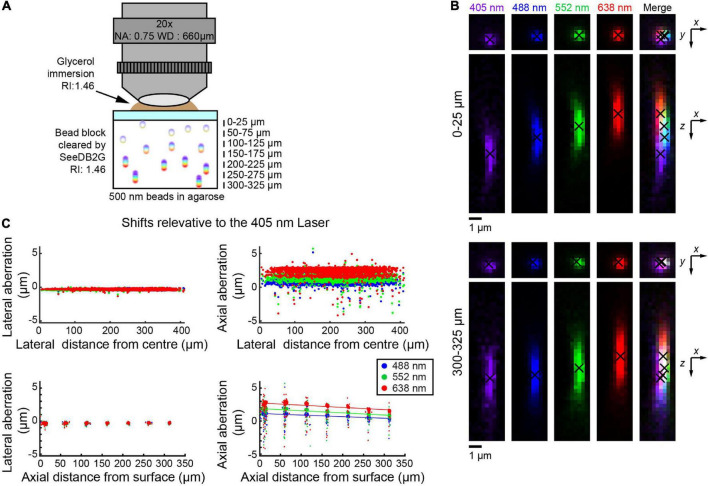
Measuring chromatic aberrations with a multi-immersion objective lens (Leica 20×). **(A)** Fluorescent beads (500 nm) were embedded in agarose gel and then cleared with SeeDB2G (RI, 1.46). The agarose sample was mounted with a 0.17 mm thick coverslip. Glycerol (RI, 1.46) was used for immersion. **(B)** High-magnification images of fluorescence beads at representative depths (MIPs). Centroid positions are indicated by black crosses. **(C)** Lateral and axial chromatic aberrations at 488, 552, and 638 nm were determined as shifts relative to the 405 nm laser. Positive axial aberration values indicate that the bead is higher than it appears in the 473 nm image. Depth-dependent axial aberrations were observed.

We also tested the multi-immersion 20× objective lens with SeeDB2G, using 500 nm beads (Figure 3A). Images were acquired at representative depths. Similar to the two previous setups, lateral aberrations were minimal, but axial aberrations were more prominent (Figure 3B). With this objective lens, the trend was that the aberration decreases as the depth increases. The trends were again linear and so could be fit with linear regression lines (Figure 3C). Furthermore, the trends between all the different excitation lasers are linear, i.e., any laser could be used as a reference.

### Correction of Chromatic Aberrations in Biological Samples Using a Reference Sample

**FIGURE 4:**
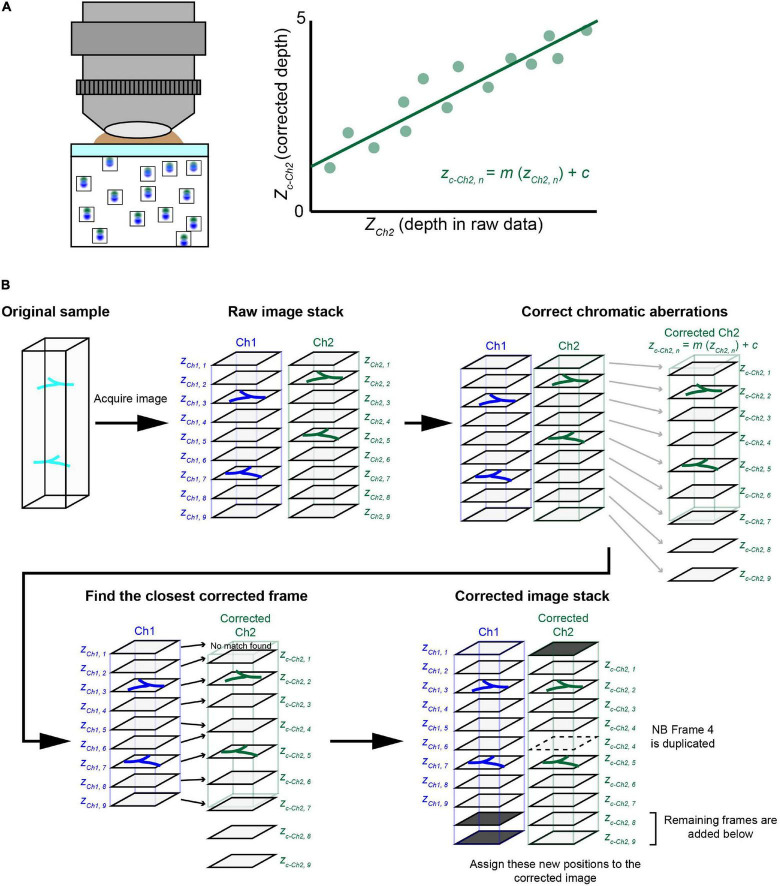
The chromatic aberration correction pipeline. **(A)** We developed a chromatic aberration correction pipeline. After calculating the depth-dependent axial chromatic shifts, we assigned a correct frame of channel 2 (Ch2) for each frame of a reference channel 1 (Ch1). In one approach, we used a reference bead sample to obtain necessary parameters for the correction. We used agarose gel containing fluorescent beads to determine the relationship between depth and axial aberrations. We then obtained a regression equation to determine the corrected depth for Ch2, which should match that of Ch1. Another approach using guide stars within the sample is detailed in Figure 6. **(B)** The *post hoc* correction pipeline. After taking volumetric fluorescence images for Ch1 and Ch2, we assigned *z* depths (actual movement of the objective) for both Ch1 and Ch2. Due to chromatic aberrations, the *z* position for Ch2 (*z*_*Ch*2, *n*_) is shifted relative to Ch1 (zCh1, n). We therefore calculated “corrected” *z* positions for Ch2 (*z*_*c*–*Ch*2, *n*_), which should be consistent with *z*_*Ch*1, *n*_. Then, for each of the Ch1 frames, we assigned a Ch2 frame with the closest *z*_*c*–*Ch*2, *n*_ value. In the new image stack after this correction, the chromatic aberration will be fully cancelled. Occasionally, some Ch2 frames will be deleted or duplicated.

Our evaluation with fluorescent beads showed almost negligible lateral aberrations and a linear relationship for axial aberrations in all three objectives. This led us to ask whether we could calculate the expected aberration and then correct volumetric images of biological samples labeled with multiple fluorophores. We extracted the linear regression equations for all the excitation lasers with respect to a reference excitation laser (Figure 4A). These equations can then be applied to other volumetric images taken under the same conditions (Figure 4B).

Figure 4B explains the procedure for the *post hoc* correction pipeline of chromatic aberration based on the regression equation from reference data. Here, we considered fluorescence image stacks taken with two channels, namely, Ch1 and Ch2. We aimed to align corrected Ch2 frames for Ch1 frames based on the regression equation. First, we calculated correct *z* position (*z*_*c*–*Ch*2, *n*_) for each frame of Ch2 based on the original *z* position (*z*_*Ch*2, *n*_). We then assigned the closest frame of Ch2 to each frame of Ch1 (*z*_*Ch*1, *n*_) based on its *z* position (*z*_*c*–*Ch*2, *n*_). We simply picked up a Ch2 frame whose corrected *z*-position (*z*_*c*–*Ch*2, *n*_) is closest to that of a Ch1 frame (*z*_*Ch*1, *n*_). As the aberration is not constant, frames from Ch2 may be occasionally duplicated (i.e., broken lines) or deleted in this assignment process. In this way, we obtained aberration-corrected images stacks for Ch1 and Ch2 (Figure 4B). As the regressions were calculated for all possible combinations of lasers, any channel can be chosen as the reference channel.

To assess the utility of this *post hoc* correction pipeline for biological data, we used the mouse brain samples labeled with a multicolor labeling technique, namely, Tetbow (Sakaguchi et al., 2018). Tetbow plasmids were introduced into layer 2/3 neurons in the cerebral cortex using *in utero* electroporation. Similar to Brainbow, Tetbow utilized the stochastic expression of three fluorescent proteins (i.e., mTurquoise2, EYFP, and tdTomato) to produce various color combinations at sufficient brightness. Each neuron was labeled with a unique color combination, aiding the efficient tracing of neurites based on the neuron-specific color hues. However, the three fluorescent proteins were excited at different wavelengths of light. Therefore, axial chromatic aberration was inevitable, making it difficult to precisely merge all the three channels across all the depths. The correction of axial chromatic aberration was important to accurately represent the color combinations in multicolor volumetric images.

**FIGURE 5:**
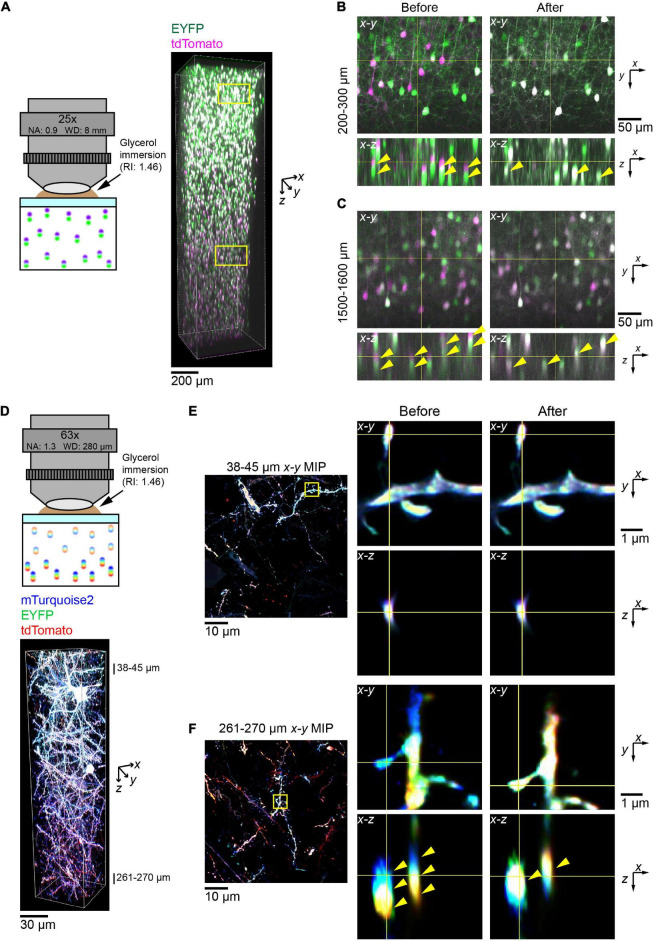
Correction of chromatic aberration in a volumetric fluorescence image of the brain based on beads reference. **(A)** The *post hoc* correction of chromatic aberration for a 25× objective lens. Fluorescence images of EYFP (excited at 473 nm) and tdTomato (559 nm) were aligned based on the bead data (from Figure 1) as described in Figure 4. Neurons in the cerebral cortex were labeled by *in utero* electroporation at E15. A 2 mm thick coronal section was cleared with SeeDB2G. Age, P25. **(B,C)** Fluorescence images before and after the correction of chromatic aberrations. Signals for EYFP and tdTomato were separated before the correction but merged after the correction. Note that more doubly labeled neurons are present after the correction (yellow arrow heads). **(D)** The *post hoc* correction of chromatic aberration for a 63× objective lens. Neurons were labeled with Tetbow (mTurquoise2, EYFP, and tdTomato), and a 300 μm thick coronal section was cleared with SeeDB2G before imaging. Age, P101. **(E,F)** Fluorescence images in the shallow and deep areas before and after the *post hoc* correction. Using bead reference, chromatic aberration was corrected for brain sample images. Left panels display *x*–*y* maximum intensity projections over the specified *z* range, with the zoomed-in area highlighted by the yellow square. Right panels display orthogonal sections before (left) and after (right) the correction. Note that three different fluorescence channels were separated before the correction but fully merged after the correction in the deep area (yellow arrowheads).

First, we attempted to correct data taken with the custom-made 25× objective lens and two excitation lasers at 473 and 559 nm (Figure 5A). As with the bead data, there were noticeable axial aberrations that can be particularly seen in the duplication of soma positions (yellow arrowheads, left panels, Figures 5B,C). However, after applying our *post hoc* corrections, we could see that the two channels were fully aligned along *z* at both shallow and deep locations. This could be seen by the position of somas (yellow arrowheads, right panels, Figures 5B,C, respectively). Then, there was a higher percentage of somas, which appeared double labeled with EYFP and tdTomato.

Next, we imaged the Tetbow-labeled cortical sample with the glycerin-immersion 63× objective lens with a higher NA (NA 1.3) (Figure 5D). Using this objective lens, we could clearly visualize fine dendritic spines and axons. However, chromatic aberration was evident for these fine structures, especially in the deep area (middle panels, Figure 5F). After *post hoc* corrections based on the bead reference data, we could then fully correct chromatic aberrations at all depths (right panels, Figures 5E,F).

### Correction of Chromatic Aberration Using Guide Stars Within a Biological Sample

**FIGURE 6:**
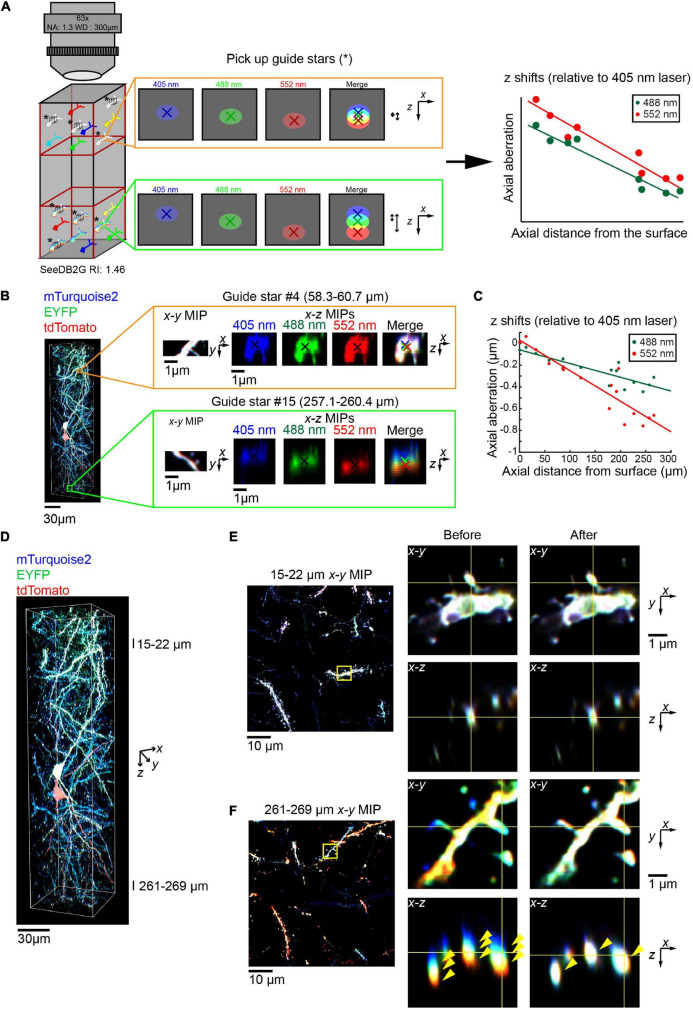
Correction of volumetric fluorescence images of the brain based on “guide star” measurement. **(A)** Schematic for the chromatic aberration correction using guide stars. Instead of beads, we can use guide stars within a biological sample to record the chromatic aberrations. Guide stars should be isolated from other objects along *x, y*, and *z* and labeled with all the fluorophores. As the regression is linear, guide stars were taken from the top and bottom third (left cartoon). The axial chromatic aberration of each guide star was then calculated using the differences in the center of mass for each channel (middle cartons). These aberrations were then used to calculate the linear regression (right cartoon). **(B)** A 63× glycerol lens was used to image a volumetric fluorescent image of a 300 μm thick coronal section containing layer 2/3 cortical pyramidal neurons labeled with Tetbow plasmids at E15, age P101. Two (out of 16) representative guide stars, showing the increasing axial chromatic aberration as the depth increases. **(C)** Linear regressions relative to the 405 nm excitation laser were calculated for both the 488 and 552 nm lasers, based on the axial aberrations of the guide stars. **(D–F)** The correction of chromatic aberration using the guide stars as a reference. The full volume **(D)** is now corrected, with fluorescence images in the shallow **(E)** and deep **(F)** areas before and after the *post hoc* correction. Left panels of **(E,F)** display *x–y* maximum intensity projections over a specified *z* range, with the zoomed in area highlighted by the yellow square. Right panels display orthogonal sections before (left) and after (right) the correction. Note that three different fluorescence channels were separated before the correction but fully merged after the correction in the deep area (yellow arrowheads).

We also examined whether we could obtain accurate parameters for regression equation from the biological sample itself, without the use of reference samples. We identified triple-labeled “guide star” objects within the sample, instead of fluorescent beads (Figure 6A, left, asterisks). When the guide star can be excited by all the different lasers, then the images with different excitations can be used to estimate the chromatic aberrations. We determined axial chromatic aberrations based on shifts in its centroid (Figure 6A, middle). We measured chromatic aberrations at multiple depth points to obtain the regression equation (right panel, Figure 6A).

We took this guide star approach for a Tetbow sample taken with the multi-immersion 63× objective lens (Figure 6B). We selected several neurite fragments at several different depths, which were excitable by all the lasers and were fully separated from other neurites along *z*. For each guide star, we determined centroid positions for each channel (Figure 6B), and the chromatic aberrations were estimated (Figure 6C). To determine an accurate number of guide stars, the *r*^2^ values for each regression were obtained (Figure 6F, *r*^2^ = 0.757 and 0. 845 for 405–488 and 552 nm, respectively). The lowest value was then used to calculate Cohen’s *f*^2^ and so to calculate the required sample size using G*Power3 (Faul et al., 2009). This suggested that for our data, 7–10 guide stars produce a power of 0.95–0.99, respectively.

Using the correction pipeline in Figure 4B and parameters from guide stars, we were able to fully correct the chromatic aberration for 63× images (Figures 6D–F). Thus, we do not always need to take images for bead reference samples.

## Discussion

Chromatic aberration is a common problem in multicolor imaging of cleared samples. Our analyses of fluorescent beads revealed that lateral chromatic aberration is almost negligible, while axial aberration is often a significant issue. Moreover, the axial aberration is often depth-dependent. Therefore, we developed a pipeline to correct the depth-dependent axial chromatic aberrations based on the reference data. Our code is freely available for use (see text footnote 1). We demonstrated the utility of our pipeline for brain samples labeled with Tetbow based on mTurquoise2 (excited at 405 nm), EYFP (excited at 473/488 nm), and tdTomato (excited at 552/559 nm). However, these corrections can be applied to any number of different excitation lasers, including those that utilize more than three excitation lasers. In connectomics, multicolor imaging provides a great opportunity to help resolve dense and large neuronal networks (Livet et al., 2007; Lichtman et al., 2008; Sakaguchi et al., 2018). The quantitative analysis of dendritic spines and synapses would require a higher precision in composing multichannel images. As tissue-clearing techniques are now broadly used for large-scale volumetric imaging in biology, our pipeline will be useful for a wide range of applications. For example, if samples are cleared with Scale or CUBIC, then the same protocol can be applied provided that the refractive indices are matched. This can be achieved by using 2,2′-thiodiethanol (Ke et al., 2013); however, the lens will still need to be designed for the immersion media.

In our first application, we could successfully obtain an aberration-corrected image stacks using reference bead data acquired separately (Figure 5). For each imaging setup (i.e., objective lens, clearing agent, immersion, and laser lines), users need to determine the parameters for calibration using fluorescent beads. However, this only needs to be performed once and then can be applied to all future images performed under those conditions. It is worth noting that the refractive index of the clearing agent must be the same for both the reference bead and the biological samples. Otherwise, the regressions may not be accurate.

In this study, we also suggested a strategy that uses the sample itself to obtain parameters for the *post hoc* correction of chromatic aberrations. If we can find appropriate guide stars within a sample that are excitable by all the lasers, we can estimate the degree of axial chromatic aberrations within the sample. We can then use the parameter to correct the chromatic aberrations of the entire volume of the sample. In high-resolution imaging, a subtle difference in imaging conditions (e.g., the refractive index of the sample) can affect the chromatic aberrations. Our second strategy with guide stars may be useful when the highest level of precision is needed. However, it should be noted that it may be difficult to find double-/triple-labeled guide stars in some samples. In that case, we may need to extend the emission range only for guide star measurement so that we can obtain target images excited by different wavelengths.

In this study, we did not correct lateral chromatic aberration; however, this may be needed for some objective lenses. Also, we applied linear regression to correct axial aberrations; however, non-linear transformation may better correct aberrations, especially when large volumes are images. In future, it may be useful to develop a more precise *post hoc* correction pipeline. For example, a deep-learning-based correction may be more useful to fix various types of aberrations based on the reference (Wang et al., 2019). Alternatively, one could use deep learning approaches from the data as a whole, training the algorithm by measuring the image similarity between each channel [e.g., SSIM, (Wang et al., 2004)]. A precise *post hoc* correction should be useful, especially when multiple tiles of images need to be stitched. To date, most of the efforts for the correction of aberration have been targeted at improving objective lenses. We anticipate that *post hoc* correction strategies have a lot more potential in future for the acquisition of aberration-free precise fluorescence images of a large volume.

## Data Availability Statement

The datasets presented in this study can be found in online repositories. The names of the repository/repositories and accession number(s) can be found below: SSBD:repository (https://doi.org/10.24631/ssbd.repos.2021.10.001); Github (URL: https://github.com/mleiwe/ChromaticAberrationCorrection).

## Ethics Statement

The animal study was reviewed and approved by Institutional Animal Care and Use Committee of Kyushu University.

## Author Contributions

ML and TI conceived the project. ML obtained reference data and wrote the code. SF obtained and imaged the brain samples. TI supervised the project. ML and TI wrote the manuscript with inputs from all authors. All authors contributed to the article and approved the submitted version.

## Conflict of Interest

The authors declare that the research was conducted in the absence of any commercial or financial relationships that could be construed as a potential conflict of interest.

## Publisher’s Note

All claims expressed in this article are solely those of the authors and do not necessarily represent those of their affiliated organizations, or those of the publisher, the editors and the reviewers. Any product that may be evaluated in this article, or claim that may be made by its manufacturer, is not guaranteed or endorsed by the publisher.

## Footnotes

1
https://github.com/mleiwe/ChromaticAberrationCorrection

2
https://doi.org/10.24631/ssbd.repos.2021.10.001

3
https://fiji.sc/
